# microRNAs expression profile related with response to preoperative radiochemotherapy in patients with locally advanced gastric cancer

**DOI:** 10.1186/s12885-018-4967-4

**Published:** 2018-10-29

**Authors:** Xiaowen Liu, Hong Cai, Weiqi Sheng, Hua Huang, Ziwen Long, Yanong Wang

**Affiliations:** 1 Department of Gastric Surgery, Fudan University Shanghai Cancer Center, 270 Dong An Road, Shanghai, 200032 People’s Republic of China; 2 Department of Oncology, Shanghai Medical College, Fudan University, Shanghai, 200032 China; 3 Department of Pathology, Fudan University Shanghai Cancer Center, Shanghai, 200032 China

**Keywords:** Gastric cancer, microRNAs expression, Response, Preoperative radiochemotherapy

## Abstract

**Background:**

It is urgent to find some biochemical markers for predicting the radiochemotherapy sensitivity. microRNAs have a huge potential as a predictive biomarker in gastric cancer. The current study aims to identify the microRNAs related to the radiochemotherapy sensitivity in gastric cancer.

**Methods:**

From April 2012 to August 2014, 40 patients with locally advanced gastric cancer were included into the clinical trial in the Fudan University Shanghai Cancer Center. The lesion specimens of 15 patients were obtained by gastroendoscopy before treatment, and the RNA was extracted. microRNAs array was used to identify the microRNAs with different expression level between sensitive group and non-sensitive group. The microRNAs identified in the array were further confirmed by TaqMan Real-time PCR.

**Results:**

2006 microRNAs were identified by microRNA array, including 302 highly expressed microRNAs and 1704 lowly expressed microRNAs between non-sensitive group and sensitive group. According to the statistical significance (*p* < 0.05) and expression level (more than twofold or less than 0.5 times), 9 microRNAs were identified. Finally, we chose 6 microRNAs like miR-16-2-3p, miR-340-5p, miR-338-3p, miR-142-3p, miR-142-5p and miR-582-5p to determine the sensitive group and non-sensitive group. TaqMan Real-time PCR confirmed the results of microRNA array.

**Conclusions:**

microRNA array can be used to select the microRNAs associated with radiochemotherapy sensitivity in gastric cancer. miR-338-3p and miR-142-3p may be promising predictive biomarkers for such patients.

**Trial registration:**

Trial Registration number: NCT03013010.

Name of registry: Phase II Study of Neoadjuvant Chemotherapy Wtih S1 + Oxaliplatin (SOX) Regimen Followed by Chemoradiation Concurrent With S-1 in Patients With Potentially Resectable Gastric Carcinoma.

Date registered: December 31, 2013.

The trial was prospectively registered.

**Electronic supplementary material:**

The online version of this article (10.1186/s12885-018-4967-4) contains supplementary material, which is available to authorized users.

## Background

Although the incidence of gastric cancer has been declining for several decades, it is still the fifth most common cancer and the third most frequent cause of cancer death [1]. Surgery is still most important treatment for gastric cancer; especially patients with gastric cancer can get a good prognosis after second station lymphadenectomy (D2). However, more than 60% of the patients were diagnosed at late stage in most of the countries, which resulted in low curative gastrectomy and dismal prognosis [2]. Preoperative chemotherapy has been proven to be effective in increasing the R0 resection in patients with locally advanced gastric cancer [3–5]. Compared to preoperative chemotherapy, preoperative radiochemotherapy can dramatically improve rates of pathologic complete regression (pCR) and R0 resection in patients with locally advanced gastric cancer [6–9]. However, only some of the patients with gastric cancer showed benefits after receiving radiochemotherapy. Therefore, the ability to predict response for preoperative radiochemotherapy may allow doctors to select patients that will most likely benefit from this therapy.

microRNAs are highly conserved, non-coding RNAs, which can regulate gene expression. It was reported that microRNAs regulated a large number of oncogenes, tumor suppressor genes, and genes associated with therapy resistance in gastric cancer and colorectal cancer [10–17]. Recently, a study showed that miR-221 and miR-222 can regulate radiosensitivity of gastric cancer cells [18]. However, there were only scattered studies reporting relationship between microRNAs and radiotherapy of gastric cancer, and most of the available studies did not include patient’s data.

The aim of this study was to identify microRNA signatures, which can predict response to preoperative radiochemotherapy in patients with locally advanced gastric cancer.

## Methods

### Patients

There were 15 patients with locally advanced gastric cancer. These patients were included in a clinical trial registered with ClinicalTrial.gov, number NCT03013010. In this trial, indication for preoperative radiochemotherapy was clinical stage III (eg, T4aN + M0, or T4bNXM0). Patients underwent laparoscopic exploration or exploratory laparotomy before receiving preoperative radiochemotherapy. Patients received one cycle of S-1 (80 mg/m^2^ per day on days 1 to 14) and oxaliplatin (130 mg/m^2^ on day 1) followed by concurrent radiation (45 Gy in 25 fractions, 5 days per week) and chemotherapy (S-1, 60 mg/m^2^ per day for five weeks), then underwent another cycle of S-1 (60 mg/m^2^ per day on days 1 to 14) and oxaliplatin (130 mg/m^2^ on day 1). Surgery was performed 6 weeks after completing radiochemotherapy. The standard D2 gastrectomy was recommended as the preference. Another four cycles of SOX were administered after surgery. The tumor response to radiochemotherapy was evaluated by two ways including clinical and pathological. Response evaluation criteria for solid tumors (RECIST) 1.1 was used for clinical response. Pathologically, patients with less than 10% residual carcinoma cells in the lesion were defined as responders. In the end, 10 patients were classified as responders, and 5 patients as non-responders. The written informed consent had been obtained from all the patients, and this trial was approved by the Ethical Committee of Fudan University Shanghai Cancer Center. The information have been detailed in a previous publication [19].

### Tissue sample

The lesion specimens of 15 patients were obtained by gastroendoscopy before radiochemotherapy, and the specimens were immediately stored in liquid nitrogen until RNA extraction.

### RNA extraction and purification

The RNA extraction, labeling, and analysis were performed by Shanghai Biotechnology Corporation. Total RNA was isolated using miRNeasy Mini Kit (Qiagen) according to the manufacturer’s protocol. RNA concentration, purity, and RNA integrity number (RIN) were measured using NanoDrop ND-1000 spectrophotometer (Peqlab, Erlangen, Germany), an Agilent 2100 Bioanalyzer and RNA 6000 Nano LabChip Kits (both Agilent Technologies, Santa Clara, CA, USA). A minimum RIN ≥ 6.0 was required for microarray analysis. Detailed methodology was from a previous publication [20].

### Labeling and hybridization

Total RNA was hybridized to Agilent Human microRNA (8 * 60 K) V19.0 chip (design ID: 46064). All chips were prepared using RNA 6000 LabChip kit. microRNA molecules in total RNA were spiked by using microRNA Spike-In Kit (Agilent Technologies). Subsequently, the spiked total RNA was treated with alkaline calf intestine phosphatase, labeling reaction was initiated with 100 ng total RNA per sample. T4 RNA ligase, which contained the cyanine 3-cytidine biphosphate (microRNA Complete Labeling and Hyb Kit; Agilent Technologies), was used to label the dephosphorylated RNA. The labeled microRNA samples were hybridized to human microRNA microarrays (Release 16.0, 8 9 60 K format; Agilent Technologies) at 55 °C for 20 h. Subsequently, each labeled slide was hybridized with 100 ng Cy3-labeled RNA using microRNA Complete Labeling and Hyb Kit (Cat # 5190–0456; Agilent technologies) in hybridization Oven (Cat # G 2545A; Agilent technologies) at 55 °C, 20 rpm for 20 h. Subsequently, slides were washed in staining dishes (Cat # 121, Thermo Shandon and Waltham, MA, US) with increasing stringency (Gene Expression Wash Buffer Kit Cat # 5188–5327; Agilent technologies). After washing the microarray slides, they were dried with acetonitrile (Sigma-Aldrich, St Louis, MO, USA). RNAs were isolated and amplified using identical conditions. All of the described steps were performed according to the manufacturer’s instructions. Detailed methodology was from a previous publication [20].

### Chip scan and data acquisition

Fluorescent signal intensities were measured on an Agilent DNA Microarray Scanner (Cat # G2565BA; Agilent Technologies) using the Scan Control A.8.4.1 Software (Agilent Technologies), and images were extracted using the Feature Extraction 10.7.3.1 Software (Agilent Technologies). Detailed methodology was from a previous publication [20].

### Data and bioinformatics analysis

A change with > 2-fold implies up-regulation of microRNA, and a change with < 0.05-fold implies down-regulation of microRNA. The fold-change value was calculated to determine the extent and direction of differential expression between sensitive group and non-sensitive group. Gene ontology (GO) was performed using DAVID bioinformatics resource. Kyoto Encyclopedia of Genes, and Genomes (KEGG) pathway analysis was conducted based on targets of the microRNAs that were predicted using TARGETMINER、miRDB、microRNA_org、TarBase and RNA22. Detailed methodology was from a previous publication [20].

### Quantitative real-time PCR (qRT-PCR)

The relative quantification of selected microRNAs was performed by qRT-PCR reaction with the KAPA SYBR FAST qPCR Kit Master Mix(2X) Universal (KK4601,KAPA)using Funglyn FTC-3000 Real-Time PCR System (Funglyn, Candan). The microRNA specific primers were designed by Primer Express software (Version 2.0, Applied Biosystems) based on the microRNA sequences that were obtained from miRbase database (http://microrna.sanger.ac.uk/). Primer sequences were listed in the Table 1. Extracted total RNA (60 ng) from samples was reversely transcribed into cDNA using EasyScriptTM Synthesis Kit(ABM). Each reaction was performed in a 20 μ l volume system that contained 2 μ l cDNA, 0.4 μ l of each primer and 10 μ l 2 × QuantiTect SYBR Green PCR Master Mix (Qiagen). U6 was used as a stable endogenous control for normalization. All reactions were carried out in triplicate. The relative expression levels of microRNAs were calculated by the 2^−△△Ct^ method.

**Table 1:** RT-PCR primer sequences used in microRNAs validation

microRNAs	primer sequences
miR-142-5p RT	CTCAACTGGTGTCGTGGAGTCGGCAATTCAGTTGAGAGTAGTGC
miR-142-5p F	ACACTCCAGCTGGGcataaagtagaaagcac
miR-142-3p RT	CTCAACTGGTGTCGTGGAGTCGGCAATTCAGTTGAGTCCATAAA
miR-142-3p F	ACACTCCAGCTGGGtgtagtgtttcctacttta
miR-338-3p RT	CTCAACTGGTGTCGTGGAGTCGGCAATTCAGTTGAGCAACAAAA
miR-338-3p F	ACACTCCAGCTGGGtccagcatcagtgatttt
miR-340-5p RT	CTCAACTGGTGTCGTGGAGTCGGCAATTCAGTTGAGAATCAGTC
miR-340-5p F	ACACTCCAGCTGGGttataaagcaatgagact
miR-16-2-3p RT	CTCAACTGGTGTCGTGGAGTCGGCAATTCAGTTGAGTAAAGCAG
miR-16-2-3p F	ACACTCCAGCTGGGccaatattactgtgctgc
hsa-miR-582-5p RT	CTCAACTGGTGTCGTGGAGTCGGCAATTCAGTTGAGAGTAACTG
hsa-miR-582-5p F	ACACTCCAGCTGGGttacagttgttcaaccagt
hsa-u6	CTCGCTTCGGCAGCACA
hsa-u6	AACGCTTCACGAATTTGCGT

### Protein-protein interactions (PPI) network analysis

A number of mRNAs were found in the interaction analysis between microRNAs and mRNA. The PPI analysis was performed to find out the key proteins. The selected targeted genes were put into the STRING (Search Tool for the Retrieval of Interacting Genes) database (http://string-db.org/). STRING is a meta resource, which collect most of the available information on protein–protein associations.

### Statistical analysis

Receiver-operating characteristic (ROC) curve was performed to determine the specificity and sensitivity of identified microRNA. ROC analysis was performed using MedCalc (version 10.4.7.0; MedCalc, Mariakerke, Belgium) software. Area under the ROC curve (AUC) was calculated as an accuracy index for evaluating the diagnostic performance of selected miRNA. The 95% confidence interval (CI) was used to show statistical significance.

## Results

### Patients characteristics

Fifteen patients were chosen in this study, 10 patients were classified as responders, and 5 patients as non-responders. The median age was 61 years (range 43 to 71 years). Participants comprised 12 men and 3 women. 5 patients had tumors in the upper third of stomach, 3 patients had tumors in the middle third, and 7 patietns had tumors in the lower third. The clinical T stage of all patients was T4 (Table 2).

**Table 2:** Patient characteristics

	Patient	Age	Tumor location	cT	cN	ypT	ypN	Clinical reponse
Responders	1	50–59	Antrum	4a	3	1b	3	SD
	2	60–69	Cardia	4b	2	4a	2	PR
	3	40–49	Antrum	4a	2	3	1	PR
	4	50–59	Corpus	4b	3	0	0	PR
	5	60–69	Corpus	4a	1	0	0	PR
	6	60–69	Corpus	4a	1	3	1	PR
	7	60–69	Antrum	4b	2	0	0	SD
	8	40–49	Antrum	4a	3	3	1	SD
	9	60–69	Cardia	4b	2	3	2	PR
	10	40–49	Cardia	4b	2	4a	2	SD
Non-responders	1	60–69	Antrum	4a	2	–	–	PD
	2	70–79	Cardia	4a	2	–	–	PD
	3	50–59	Antrum	4b	3	–	–	PD
	4	60–69	Antrum	4a	3	–	–	PD
	5	60–69	Cardia	4b	2	4a	2	SD

- Without receiving gastrectomy

### Screening of differentially expressed microRNAs and clustering analysis

**Fig. 1:**
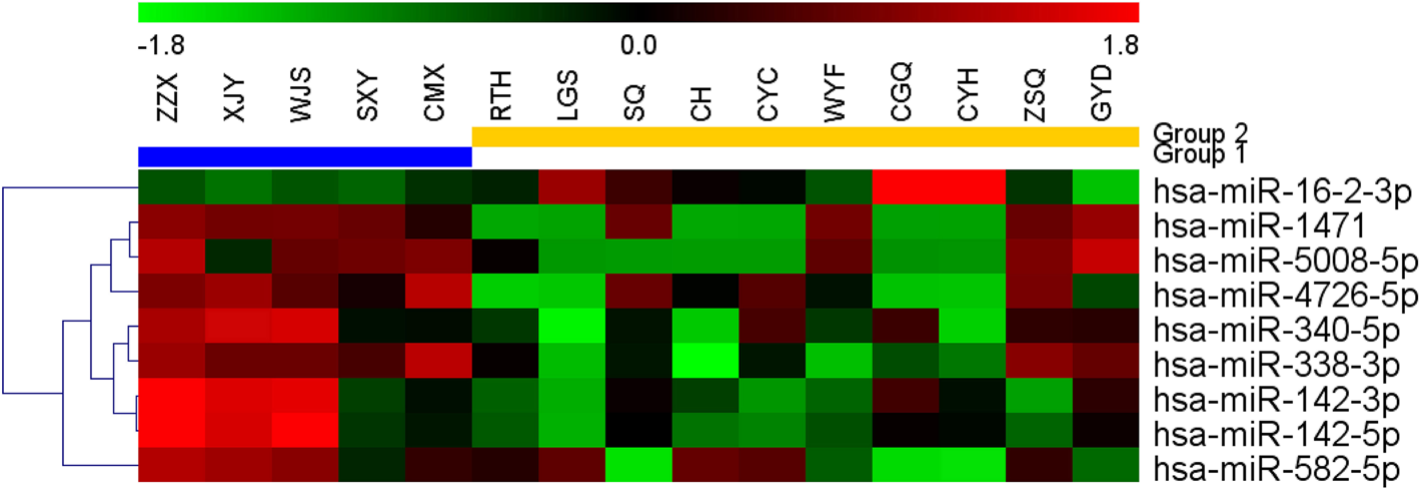
Hierarchical clustering analysis for the selected differentially expressed microRNAs. The horizontal axis represents the serum samples from gastric cancer patient with radiochemotherapy sensitive (group 2) (RTH, LGS, SQ, CH, CYC, WYF, CGQ, CYH, ZSQ and GYD) and non-sensitive group (NC) controls (group 1) (ZZX, XJY, WJS, SXY and CMX). The microRNA names are shown on the right vertical axis. Colored bars indicate the range of fold changes

To identify differentially expressed microRNAs between sensitive and non-sensitive groups, the human microRNA expression was profiled using human microRNA microarray, which contained a total of 2006 human microRNAs. Out of these 2006 microRNAs, 20 were down-regulated and 1342 were up-regulated significantly(*P*-value<0.05)in non-sensitive group compared with sensitive group. After applying a stringent filtering criteria (adjusted *P*-value<0.05, fold change ≥2 or ≤ 0.5), we identified 9 microRNAs (8 up-regulated and 1 down-regulated) (Table 3). Furthermore, hierarchical clustering analysis showed the gastric cancer samples could be well classified into non-sensitive group and sensitive group according to the expression levels of these 9 microRNAs (Fig. 1).

**Table 3:** Top 9 differential expressed microRNAs in gastric cancer patient samples between sensitive group (group 1) and control (group 2)

microRNAs	p-values	foldchange	Fold-change(abs)	Mean signal Group1	Mean signal Group2
miR-16-2-3p	0.030522	0.129112	down	−1.7468175	−0.08151509
miR-1471	0.009907	2.05241	up	2.59558034	−0.73839157
miR-5008-5p	0.026809	2.0561	up	1.39124832	−1.27935714
miR-340-5p	0.029143	2.415658	up	4.68734292	3.38271213
miR-338-3p	0.001258	2.694252	up	5.72245534	3.88186443
miR-142-3p	0.045334	2.877809	up	9.2813758	7.91703863
miR-4726-5p	0.004758	3.50025	up	1.86259976	−0.90969894
miR-142-5p	0.043532	3.828431	up	6.91469084	5.27306082
miR-582-5p	0.029775	4.116054	up	3.53336191	0.457117246

### Validation of target microRNAs

**Fig. 2:**
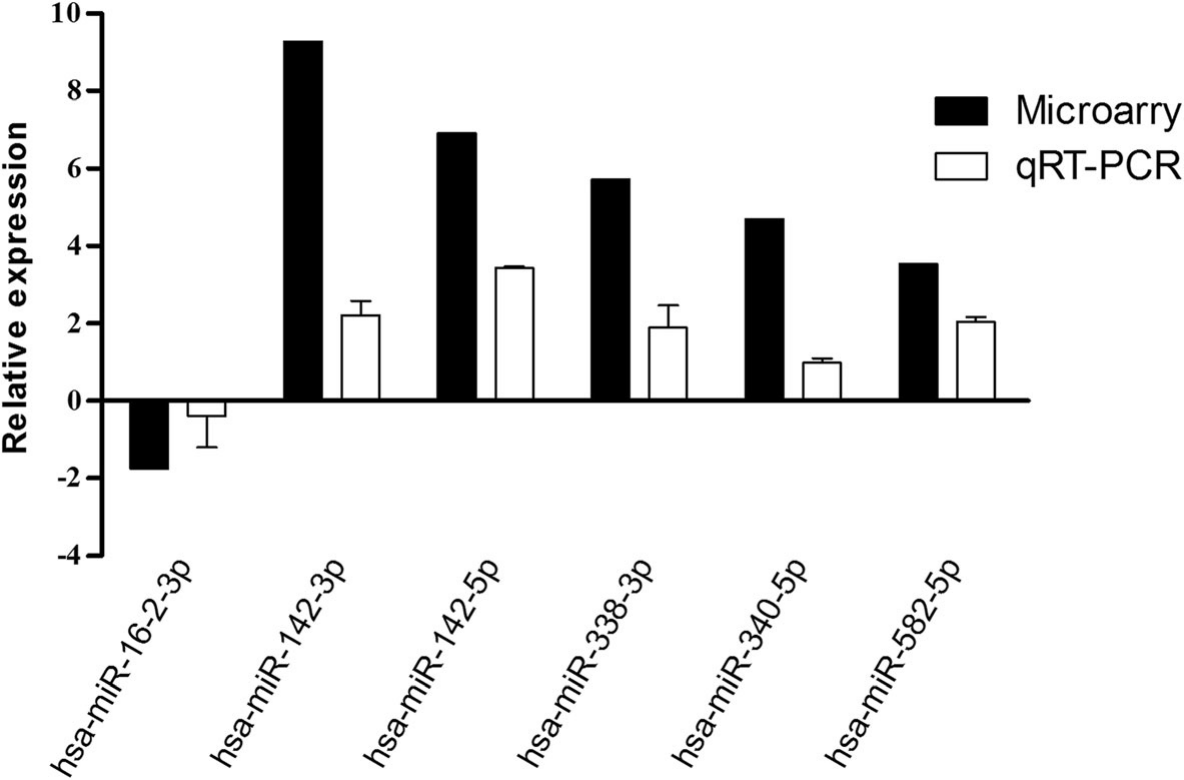
Validation of selected microRNAs by qRT-PCR. miR-12-3p, miR-142-5p, miR-338-3p, miR-340-5p, miR-582-5p and miR-16-2-3p were measured in 5 non-sensitive and 10 sensitive gastric cancer patient samples

Six microRNAs (miR-16-2-3p, miR-340-5p, miR-338-3p, miR-142-3p, miR-142-5p and miR-582-5p), which showed average signal value equal or greater than 3, were selected for further validation by qRT-PCR assay. We found that all of the 6 microRNAs showed the same change patterns as shown in microarray analysis, miR-12-3p, miR-142-5p, miR-338-3p, miR-340-5p and miR-582-5p were up-regulated, and miR-16-2-3p was down-regulated (Fig. 2).

### Functional and pathway enrichment analysis

The online tools such as TARGETMINER、miRDB、microRNA_org、TarBase, and RNA22 were used to analyze the target genes of 5 up-regulated microRNAs and 1 down-regulated microRNA. The number of predicted target genes for miR-142-3p, miR-142-5p, miR-16-2-3p, miR-338-3p, miR-340-5p and miR-582-5p was 75, 200, 60, 203, 154 and 134 respectively.

By using GO analysis of the predicted targets, we found that a large group of genes was linked with protein binding (253), zinc ion binding (112), transcription/DNA-dependent (97), protein phosphorylation (28) and so on. Pathway enrichment analysis showed that MAPK signaling pathway, Dopaminergic synapse, Endocytosis, and Glutamatergic synapse were mostly related to these 6 microRNAs (Additional file 1: Figure S1).

### A microRNA-target gene regulatory interaction network

We constructed association networks between microRNAs and the target mRNAs and visualized the target genes controlled by various IR responsive microRNA with Cytoscape. The complexity associated with microRNA interactome was shown in Additional file 2: Figure S2. The microRNA: mRNA association network provided nodes and connections between many microRNAs and the target mRNAs. This network showed the overlapping mRNA targets for the miR-142-3p, miR-142-5p, miR-16-2-3p, miR-338-3p, miR-340-5p and, miR-582-5p. We found that a number of microRNAs could work together to downregulate several genes.

### Protein-protein interaction (PPI) network

By using STRING10, we found that the interaction existed in total 112 proteins, which together formed the target gene interaction network. The network consists of 112 nodes and many lines, in which nodes represent proteins, and lines represent the types of evidence for the association. Results showed that MTOR, KDM6A, PTPN23, WASL, EZH2, HGS, KAT2B, and WWP1 played important roles in maintaining stability in the network (Additional file 3: Figure S3).

### Diagnostic utility of potential microRNAs

**Fig. 3:**
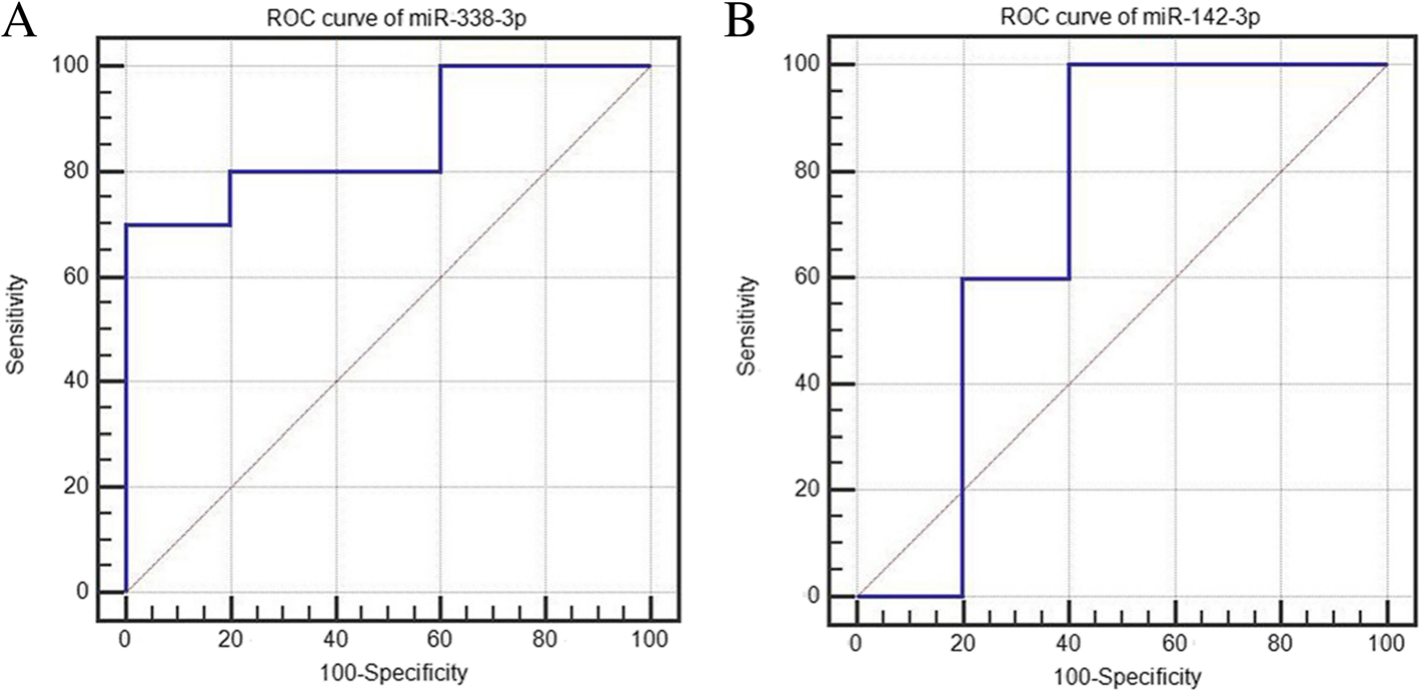
Receiver operating characteristic curve analysis for radiochemotherapy sensitive diagnosis. **a** miR-1338-3p, (**b**) miR-142-3p; AUC: area under the curve. ROC curve analysis was used by Medcalc

The diagnostic utility of the 6 microRNAs (miR-142-3p, miR-142-5p, miR-16-2-3p, miR-338-3p, miR-340-5p and miR-582-5p) were evaluated in 15 gastric cancer patient samples. The AUC of miR-338-3p and miR-142-3p was 0.86 (95% CI, 0.587–0.981; sensitivity = 70%, specificity = 100%) and 0.72 (95% CI, 0.436–0.914; sensitivity = 100%, specificity = 60%), respectively, which suggested miR-338-3p and miR-142-3p might have the potential to be predictive biomarkers of radiochemotherapy (Table 4, Fig. 3).

**Table 4:** Receiver operating characteristic curve analysis for six microRNAs in 15 gastric cancer patient samples

	miR-16-2-3p	miR-142-3p	miR-142-5p	miR-338-3p	miR-340-5p	miR-582-5p
Area(AUC)	0.54	0.72	0.68	0.86	0.52	0.58
95% confidence interval	0.271 to 0.792	0.436 to 0.914	0.396 to 0.890	0.587 to 0.981	0.255 to 0.777	0.305 to 0.822
*P* value	0.8028	0.2681	0.3754	0.0003	0.9181	0.7194
Sensitivity	50	100	100	70	70	90
Specificity	80	60	60	100	60	60

## Discussion

In many countries, most of the gastric cancer patients were diagnosed at advanced stage. According to National Comprehensive Cancer Network (NCCN) [21], patients with T2 and further stage or lymph node metastasis are recommended to receive preoperative radiochemotherapy. Nonetheless, the short-term and long-term effects might be obviously different in different patients who received preoperative radiochemotherapy. This phenomenon indicated that the sensitivity to radiochemotherapy may also interrelated with tumor itself. At present, there are still no clinical and molecule biomarkers for predicting the sensitivity to radiochemotherapy in patients with gastric cancer. Therefore, the current study aims to find out microRNAs, which can be used as predictive biomarkers for radiochemotherapy.

microRNAs can regulate expression of genes at post-transcriptional level. microRNAs played different roles by targeting to different genes. By binding to genes related to radiochemotherapy sensitivity, microRNAs will affect the sensitivity of tumor. Weidhaas et al. [16] firstly reported that microRNAs were related to radiotherapy sensitivity in lung cancer cell. Some other studies showed that microRNAs were related to radiotherapy sensitivity in prostate cancer, breast cancer, and lung cancer [22–24]. It is reported that several microRNAs were related with radiosensitivity of gastric cancer cell lines [18, 25]. Zhi Z et al. [18] reported that suppressing the expressions of miR-221 and miR-222 could improve the radiosensitivity of gastric cancer cell line SGC7901. Liu et al. [25] found miR-375 affected the radiosensitivity of gastric cancer to a certain extent. However, all these studies were based in vitro test, which limited the clinical value of these identified microRNAs. Meanwhile, we did not observe these microRNAs in our results, which may be attributed to the differences in sample. In present study, gastric cancer tissue samples were used to identify chemoradiotherapy-related microRNAs. We found that the expression levels of 9 microRNAs were significantly different between two groups (*P* < 0.05, Fold-change ≥2 or ≤ 0.5). We chose miR-16-2-3p, miR-142-3p, miR-142-5p, miR-338-3p, miR-340-5p, miR-582-5p for further research. qRT-PCR results revealed that miR-142-3p, miR-142-5p, miR-338-3p, miR-340-5p, miR-582-5p were up-regulated distinctly in non-sensitive group. On the contrary, miR-16-2-3p was down-regulated in non-sensitive group. The qRT-PCR result were completely consistent with microarray result. Furthermore, the diagnostic utility of these six microRNAs were analyzed in 15 gastric cancer patient samples, and results showed that miR-338-3p and miR-142-3p might be potential biomarkers for predicting chemoradiotherapy sensitivity. Our results may suggest potentially useful new biomarkers in the prediction of radiochemotherapy response in patients with gastric cancer. There are still some limitation in present study. Firstly, our study included a small sample size in a microarray study, which may limit the accuracy of prediction. Secondly, the results were not validated in a large cohort.

To further investigate the mechanisms of these microRNAs in gastric cancer, 826 potential target genes were obtained by online tools. GO analysis revealed that a large group of genes were linked with protein binding, zinc ion binding, transcription/DNA-dependent, protein phosphorylatio and so on. Pathway enrichment analysis indicated that MAPK signaling pathway were mostly related to these six significantly differential expressed microRNAs. Although the mechanisms that these microRNAs affect chemoradiotherapy sensitivity in gastric carcinoma were totally unclear, some studies showed these microRNAs played important role in other cancers. It has been reported that miR-338-3p could suppress migration and invasion of intestinal cancer cell [26] and abnormal expression of miR-338-3p would increase the risk of esophagus cancer [27]. miR-142-3p was over-expression in bromocriptine-resistant prolactinoma [28], and its expression level was related with generation of acute leukemias [29]. In esophagus cancer, the expression level of miR-142-3p might affect histological differentiation and prognosis [30]. Zhang et al. [31] revealed that miR-142-5p might be a potential biomarker for predicting recurrence of gastric cancer. miR-558-5p could promote proliferation of prostatic cancer cell [32] and maintain hyperplasia of glioblastoma [33]. Uchino et al. [34] found that over-expression miR-582-5p could suppress proliferation and invasion of bladder cancer. So far, there haven’t been reports about miR-16-2-3p and miR-340-5p in cancer. Meanwhile, there are little studies about the functions of above microRNAs in treatment sensitivity of tumor, as well as in chemoradiotherapy sensitivity of gastric carcinoma.

## Conclusions

In conclusion, studies focusing on chemoradiotherapy sensitivity of gastric carcinoma are relatively little. This study has screened out a small fraction of microRNAs related with it. Results revealed that miR-142-3p, miR-142-5p, miR-338-3p, miR-340-5p, miR-582-5p were up-regulated in insensitive group, compared to sensitive group. On the contrary, miR-16-2-3p was down-regulated in insensitive group. These microRNAs might be biomarkers for sensitivity of radiochemotherapy in gastric cancer. In our future study, sensitivity and specificity of predicting radiochemotherapy sensitivity of microRNAs will be investigated in large-scale patients.

## Additional files

Additional file 1: **Figure S1.** GO analysis for six differentially expressed mRNAs and Pathway analysis based on the KEGG database. (A) GO analysis according to biological process ranked by enrichment score (−log10 (*p* value)). (B) The X-axis represents −log10 (*p* value) mapping to the given pathway. The Y-axis represents the pathways based on the decreasing order of −log10 (*p* value). The significance of each pathway was estimated based on FDR corrected *p*-values (*p*-value < 0.05). Statistic significance was analyzed by Fish exact test. (JPG 473 kb)

Additional file 2: **Figure S2.** Visualization of microRNAs and their associated target genes network with Cytoscape. The interaction network shows nodes and connections between microRNAs and the target genes. The red nodes represent the microRNA and the green/blue nodes represent its targeted gene. (JPG 2183 kb)

Additional file 3: **Figure S3.** Protein-protein interaction (PPI) network for the predicted target genes of differentially expressed microRNAs. (JPG 630 kb)

## Abbreviations

AUC: Area under the ROC curve
CI: Confidence interval
D2: Second station lymphadenectomy
GO: Gene ontology pathway analysis
KEGG: Kyoto Encyclopedia of Genes and Genomes pathway analysis
NCCN: National Comprehensive Cancer Network
pCR: Pathologic complete regression
PCR: Polymerase Chain Reaction
PPI: Protein-Protein Interactions Network Analysis
R0: No residual tumor
RECIST: Response evaluation criteria for solid tumors
ROC: Receiver-operating characteristic
SOX: S-1 and oxalipatin
STRING: Search Tool for the Retrieval of Interacting Genes database
