# Supplementary figures and images for: microRNAs expression profile related with response to preoperative radiochemotherapy in patients with locally advanced gastric cancer
Source: BMC Cancer. 2018 Oct 29;18:1048. doi: 10.1186/s12885-018-4967-4 (PMC6206758; doi:10.1186/s12885-018-4967-4)

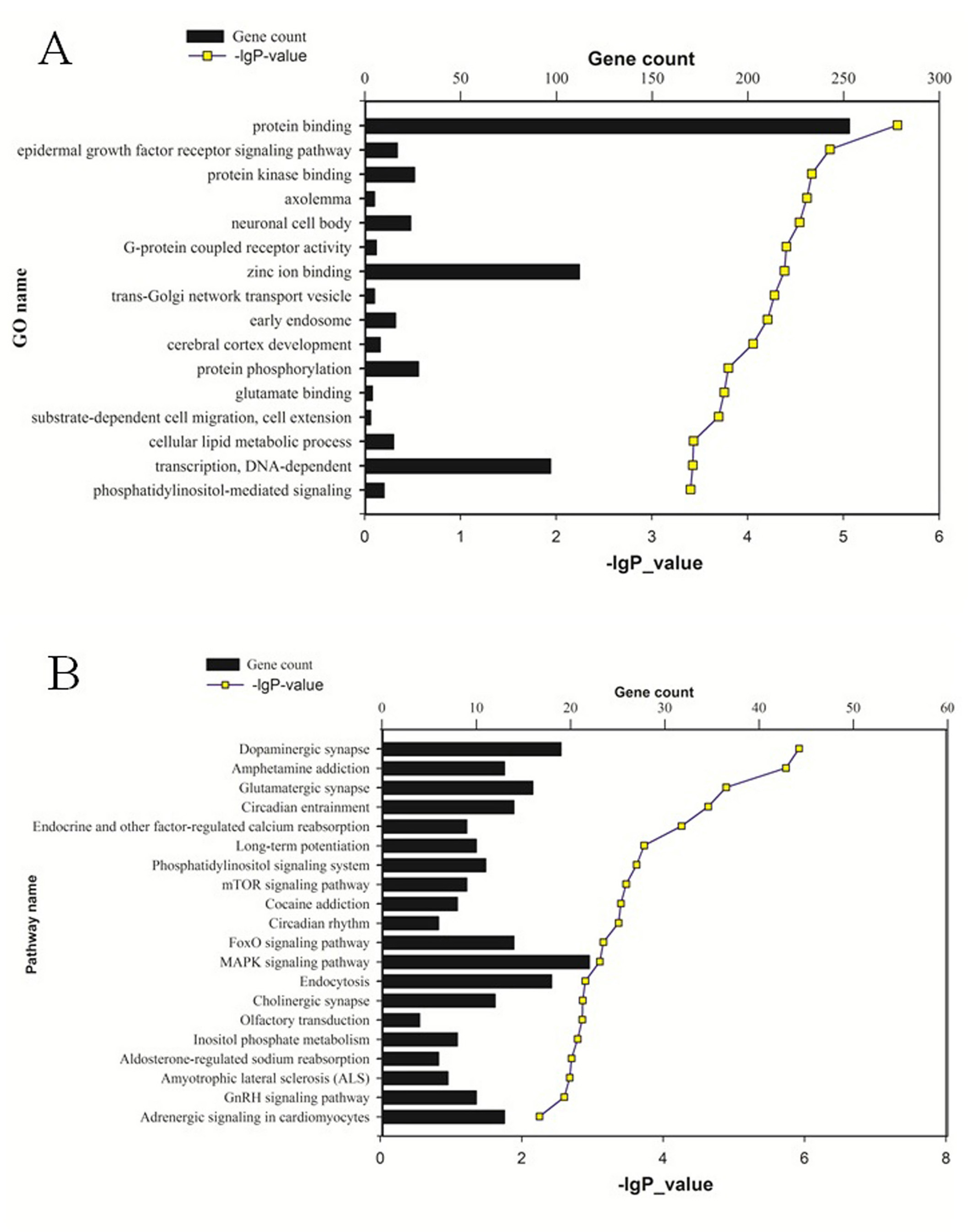

Supplement: Supplementary file 1 — Figure S1. GO analysis for six differentially expressed mRNAs and Pathway analysis based on the KEGG database. (A) GO analysis according to biological process ranked by enrichment score (−log10 (p value)). (B) The X-axis represents −log10 (p value) mapping to the given pathway. The Y-axis represents the pathways based on the decreasing order of −log10 (p value). The significance of each pathway was estimated based on FDR corrected p-values (p-value < 0.05). Statistic significance was analyzed by Fish exact test. (JPG 473 kb) [file 12885_2018_4967_MOESM1_ESM.jpg]

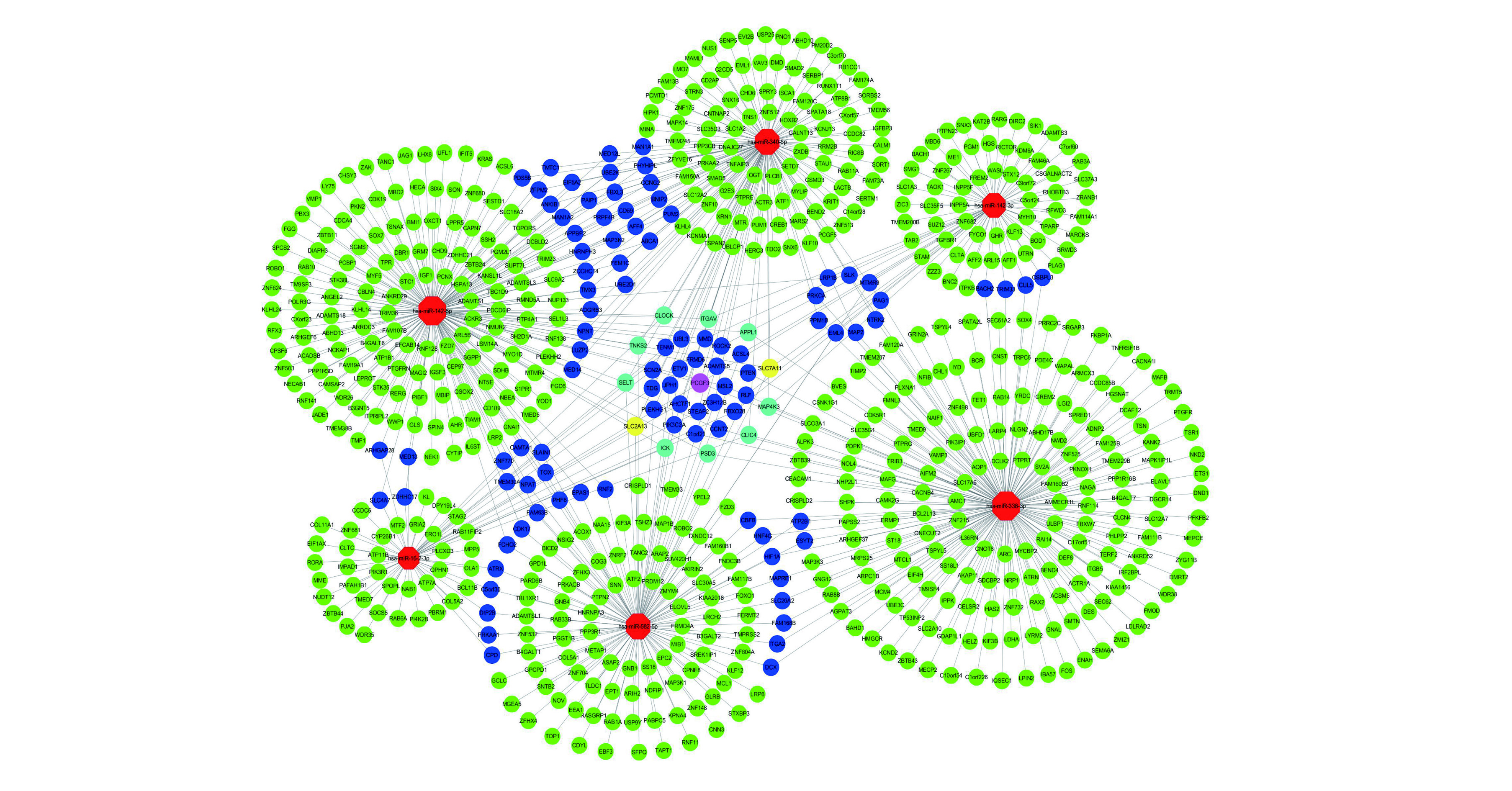

Supplement: Supplementary file 2 — Figure S2. Visualization of microRNAs and their associated target genes network with Cytoscape. The interaction network shows nodes and connections between microRNAs and the target genes. The red nodes represent the microRNA and the green/blue nodes represent its targeted gene. (JPG 2183 kb) [file 12885_2018_4967_MOESM2_ESM.jpg]

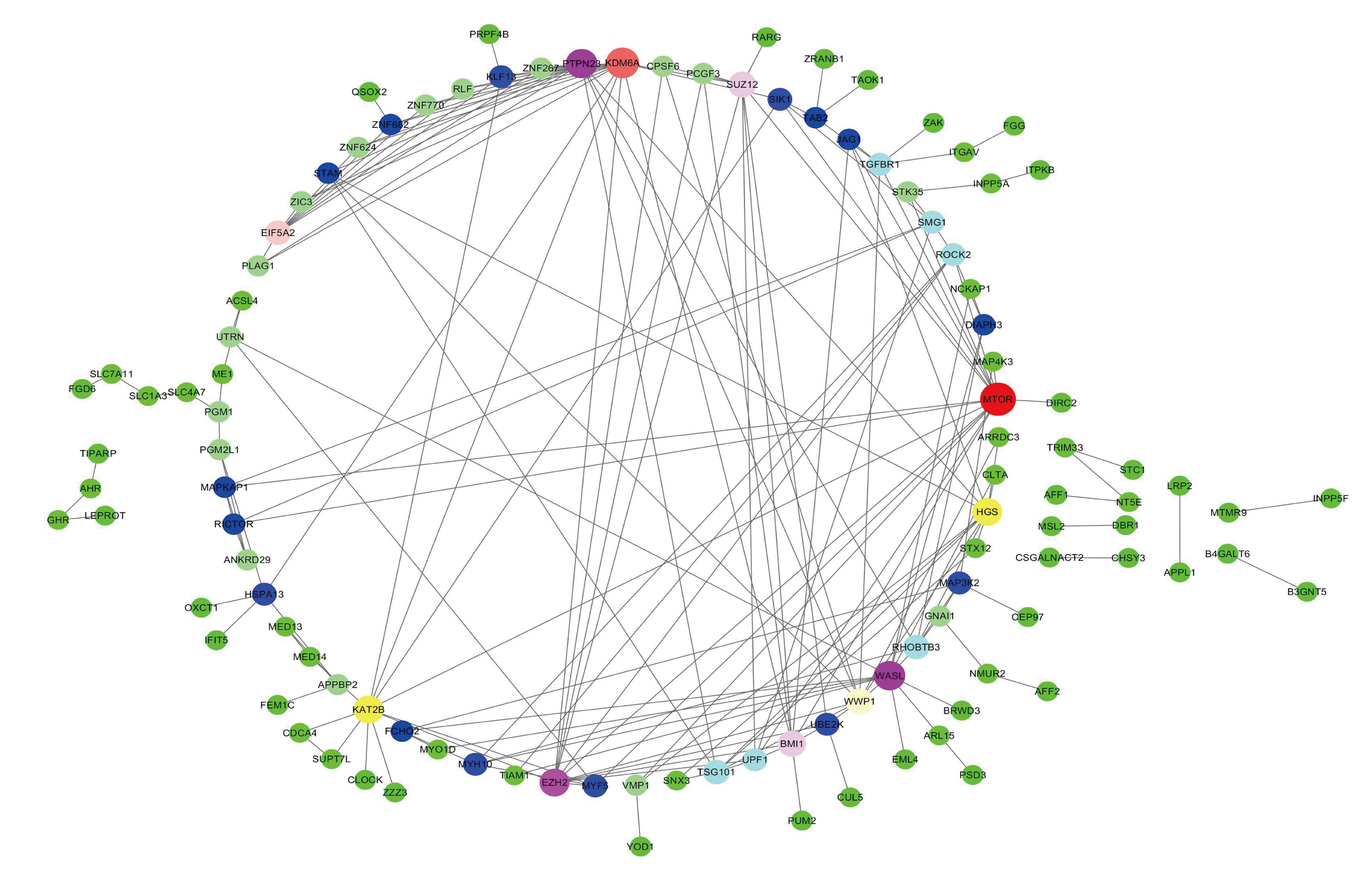

Supplement: Supplementary file 3 — Figure S3. Protein-protein interaction (PPI) network for the predicted target genes of differentially expressed microRNAs. (JPG 630 kb) [file 12885_2018_4967_MOESM3_ESM.jpg]
